# Potentiation of a novel palladium (II) complex lethality with bee venom on the human T-cell acute lymphoblastic leukemia cell line (MOLT-4)

**DOI:** 10.1186/1678-9199-19-25

**Published:** 2013-10-03

**Authors:** Zahra Safaeinejad, Mohammad Nabiuni, Zahra Nazari

**Affiliations:** 1 Department of Cell and Molecular Biology, Faculty of Biological Sciences, Kharazmi University, Tehran, Iran

**Keywords:** Apoptosis, Bee venoms, Cytotoxicity, MOLT-4 cell line, Pd (II) complex

## Abstract

**Background:**

Although honeybee venom (BV) has been reported to induce apoptosis in different types of cancerous cells, its synergistic effects with customary anti-cancer drugs remain largely unknown. In the present study, we evaluated the cytotoxic effect of BV alone (as a natural product) and the synergistic cytological effects of this component in combination with [Pd (bpy) (Pi-Pydtc)]NO_3_ – a novel palladium complex on human T-cell lymphoblastic leukemia cells. To investigate the cytotoxic effect of the BV alone and in combination with palladium complex on MOLT-4 cells MTT assay was performed. In order to determine the apoptotic effects of BV separately and in combination with Pd (II) complex on these cells and its ability to induce apoptosis, morphological examination, flowcytometric analysis and caspase-3 colorimetric assay were done.

**Results:**

We found that BV induced morphological changes, namely nuclear shrinkage, and inhibited MOLT-4 cell proliferation; both effects were dose- and time-dependent. Flow cytometry by Annexin-V antibody demonstrated that BV induced apoptosis in MOLT-4 cells. Furthermore, BV induced apoptosis independently of caspase-3 in these cells. In addition, we proved a clear synergistic effect of BV on [Pd (bpy) (Pi-Pydtc)]NO_3_. The apoptotic pathway activated by BV in combination with Pd complex was caspase-3-dependent.

**Conclusions:**

These observations provide an explanation for the anti-proliferative properties of BV, and suggest that this agent may be useful for treating lymphoblastic leukemia alone or in combination with chemotherapy drugs pending further investigations on animal models as preclinical tests.

## Background

Bee venom is a natural substance that contains only 0.1 μg of dry venom [1]. The dry venom has a very complex mixture of such active peptides as melittin, apamin and adolapine, enzymes including hyaluronidase and phospholipase A2, biologically active amines such as histamine and epinephrine as well as non-peptide components with numerous medicinal properties [2]. Melittin, a hemolytic and strong cardiotoxic peptide, is the major active ingredient of BV. This main constituent of bee venom has been reported to induce apoptosis, and to produce anti-tumor effects [3,4]. Melittin, which makes up 50-60% of the dry venom, is a low-molecular-weight protein (2846.46 Da), which is composed of 26 amino acids. It is found as a tetramer in the poison sac of the bee, but when influencing a cell, it acts as a monomer [5].

BV has been used as a traditional medicine to treat various diseases such as arthritis, rheumatism, back pain and skin diseases [2]. Besides, recent studies have reported that BV causes growth arrest and exerts cytotoxic effects on various types of cancerous cells [6-11]. The cytotoxic effects mediated through the activation of PLA2 by melittin have been suggested to be the critical mechanism for the anti-cancer activity of BV [12].

It is well documented that induction of apoptosis is the most effective strategy by which anti-cancer agents target cancer cells [13]. Chemotherapy agents can induce apoptosis signaling through two major pathways. One is the mitochondrial (intrinsic) pathway and the other one is the death receptor (extrinsic) pathway. Cascading intrinsic pathway activation of certain molecules finally provokes activation of downstream caspase-3, which is one of the key agents of apoptosis. Activated caspase-3 cleaves a wide array of substrates, such as poly(ADP-ribose) polymerase (PARP), a DNA repair enzyme, and inevitably leads to cell death [14,15].

Cisplatin (*cis*-diammine dichloroplatinum II) is one of the most remarkable drugs which is used separately or in combination with other chemotherapy agents to treat different types of tumors [16,17]. Despite the success of cisplatin and platinum-based drugs, they have presented serious clinical side effects [18,19]. Therefore, much effort has been focused on identifying novel anti-tumor agents and examining new approaches to increase their damage to tumor cells at a lower concentration than conventional chemotherapy drugs [20].

The significant similarities between the coordination chemistry of palladium (II) and platinum (II) compounds have generated lines of research on Pd (II) complexes as anti-tumor components [21]. Recently we stated at the FAOBMB conference that [Pd (bpy) (Pi-Pydtc)]NO_3_, as a novel palladium complex designed and synthesized by our research group, exerts clear anti-tumor effects on human lymphoblastic leukemia MOLT-4 cells [22].

In the present study, we first examined the cytotoxic effect of BV on the MOLT-4 cancerous cell line, then the synergistic effects of BV and the novel Pd (II), [Pd(bpy)(Pi-Pydtc)]NO3, on these cells. This investigation employed the following techniques: MTT assay, morphological analysis, flow-cytometry assay and the caspase3 activity assay.

## Methods

### Bee venom collection and novel Pd (II) complex preparation

Venom from the Iranian honey bee (*Apis mellifera*) was prepared by placing bees on a 6-mm wire grid, which was electrically pulsed. The bees then produced venom that dropped onto a glass slide, from which it was collected and freeze-dried according to the method of Lariviere and Melzack [23], whereas the novel complex of the Pd (II) was designed and synthesized by our research group [24].

### Cell culture

The human T-cell acute lymphoblastic leukemia MOLT-4 cells were purchased from the Pasteur Institute (Tehran, Iran). Cells were maintained in RPMI-1640 medium (Gibco, UK) and supplemented with 10% fetal bovine serum (Gibco, UK), penicillin at 100 units/mL, and streptomycin at 100 μg/mL, in a humidified incubator filled with 5% CO_2_ at 37°C. The medium was replaced every 48 hours.

### MTT cytotoxicity assay

In order to determine the cytotoxic effects of BV separately and in combination with Pd (II) complex on the MOLT-4 cells, cell viability was tested by MTT (3[4, 5-dimethylthiozol-2-yl]-2,5-diphenyl tetrazolium bromide) assay. The cells were first seeded into 24-well culture plates (Nunc, Denmark) at a density of 1.0 × 10^5^ cells/mL and subsequently incubated in a humidified 5% CO_2_ environment for one hour. The cells were then treated with BV at 1, 3, 6 and 8 μg/mL for 24 and 48 hours, the concentrations chosen as a result of precipitation of the BV in the medium. Non-treated cells were used as controls.

MTT (100 μL of 5 mg/mL) (Sigma, USA) was added to each well and incubated at 37°C for four hours. The dark blue crystals were dissolved by adding 1000 μL of 0.04 M HCl/isopropanol. After an overnight incubation in darkness, optical density (O.D.) was read at a wavelength of 570 nm using a spectrophotometer. The O.D. values of the experimental groups were divided by those of the untreated control group, and the results were presented as the percentage of cell viability.

By calculating the minimum BV dosage that killed MOLT-4 cells, we exposed cells to the lowest lethal dosages of BV and Pd (II) complex simultaneously [1 μg/mL BV/0.85 μM Pd (II) complex, 3 μg/mL BV/0.85 μM Pd (II) complex and 6.3 μg/mL BV/0.85 μM Pd (II) complex] for 24 hours. Cell survival was determined as described above.

### Morphological analysis

To monitor the effect of BV alone and in combination with Pd (II) complex on MOLT-4 cells, the cells were treated with BV and BV/Pd (II) complex, then morphologically analyzed under an inverted microscope to see whether these components were able to induce condensation of their nuclei.

### Apoptosis analysis by flow cytometry

In this study, apoptosis was measured by means of a flow cytometry assay. Cells were treated with BV and BV/Pd (II) complex for 24 hours. Then, these cells were harvested and washed with PBS. After washing, the cells were resuspended in 100 μL Annexin-V (Abcam, UK) (diluted 1:100 in blocking buffer BSA/PBS 1%) and samples were incubated overnight at 4°C. Next, the cells were washed with PBS and centrifuged (2000 rpm/10 minutes); the supernatant was aspirated and cells were incubated in the dark with 50 μL fluorescein-labeled goat anti-rabbit secondary antibody (diluted 1:100 in blocking buffer BSA/PBS 1%) for 45 minutes at 37°C. Finally, 300 μL of 1% formaldehyde was added to each tube and data were analyzed by flow cytometry using a FACSCalibur and the software Cell Quest (Becton Dickinson, USA).

### Caspase activity assay

Caspase activity was determined by colorimetric assay using a caspase-3 activation kit (Abcam, UK) according to the manufacturer’s protocol. Briefly, cells were first treated with different concentrations of BV (3.15, 6.3 and 12.6 μg/mL) and BV/Pd (II) complex [0.5 μg/mL BV/0.425 μM Pd (II) complex and 1 μg/mL BV/0.85 μM Pd (II) complex], and then lysed in lysis buffer. The supernatant was collected and incubated with the supplied reaction buffer, containing dithiothreitol and substrates, at 37°C for two hours. The reaction was measured by changes in the absorbance at 405 nm using a microplate reader. The level of caspase enzymatic activity in the cell lysate was proportional to the optical absorbance, which was read with an ELISA reader (Biotech, USA).

### Statistical analyses

Statistical differences were determined by one-way analysis of variance (ANOVA), with the results expressed as mean ± standard error of the mean (S.E.M.) for three independent experiments (n = 3). Differences were considered significant for p > 0.01.

## Results

### Cell viability assay

**Figure 1:**
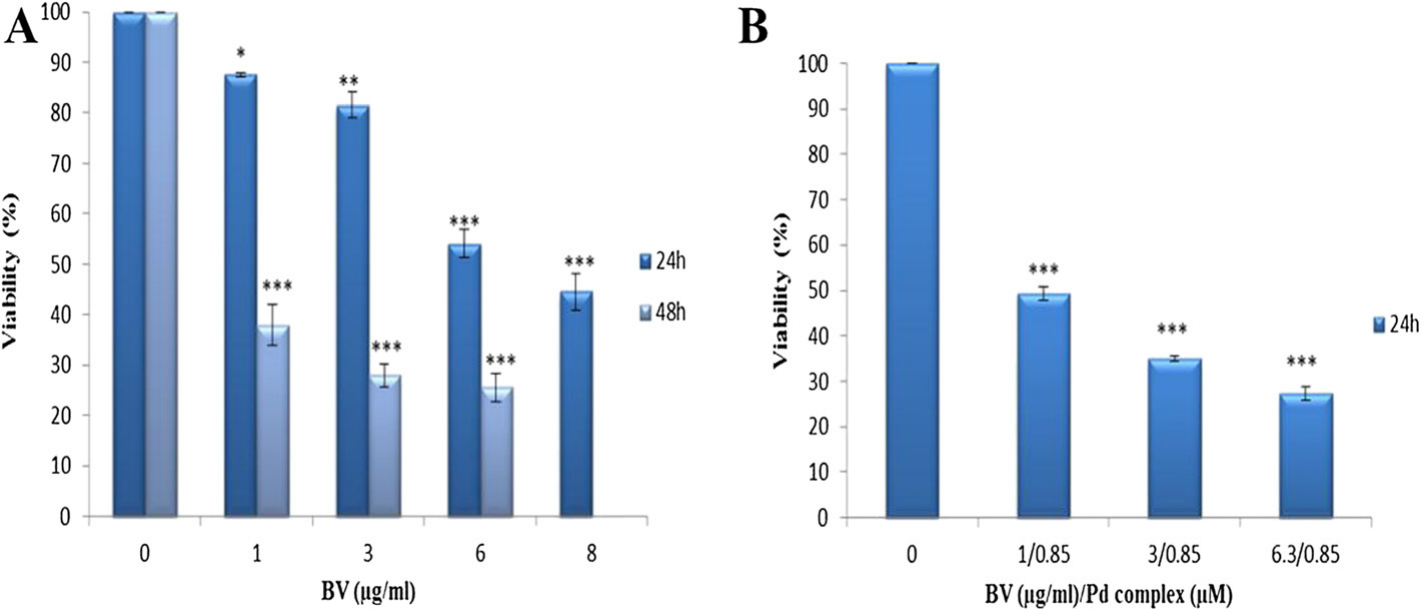
**Growth inhibition and cell death were determined via MTT assay. (A)** Dose- and time-dependent effect of BV and **(B)** dose effect of BV/Pd (II) complex on viability of MOLT-4 cells. Results are presented as mean ± S.E.M. n = 3; ** p > 0.01 and *** p > 0.001 significantly different from the control.

In order to determine the optimal dose and time of cytotoxic effect of BV alone and in combination with this novel Pd (II) complex on MOLT-4 cells, an MTT assay was performed. The cells were treated with BV at various concentrations for 24 and 48 hours and with BV/Pd (II) complex for 24 hours. The respective viabilities of cells treated with BV at concentrations of 1, 3, 6 and 8 μg/mL for 24 hours were 87.5 ± 0.500, 81.5 ± 2.500, 54 ± 2.828 and 44.5 ± 3.5 in relation to the control value. The viabilities of cells treated with BV at the concentrations of 1, 3 and 6 μg/mL for 48 hours were 38 ± 4, 28 ± 2.309 and 25.6 ± 2.728 relative to the control value, respectively (Figure 1A).

The viabilities of cells treated with BV/Pd (II) complex at concentration of 1 μg/mL BV/0.85 μM Pd (II) complex, 3 μg/mL BV/0.85 μM Pd (II) complex and 6.3 μg/mL BV/0.85 μM Pd (II) complex for 24 hours were 49.33 ± 1.435, 35 ± 0.5774, 27.33 ± 1.453 in relation to the control value, in that order (Figure 1B).

These results reveal that the cytotoxic effect of BV alone and in combination with Pd (II) complex on MOLT-4 cells is dose- and time dependent (Figure 1A and B). Based on these data, the respective 50% cytotoxic concentrations (Cc_50_) of the BV after 24 and 48 hours of incubation were 6.3 and 0.6 μg/mL. The Cc_50_ value of BV in combination with Pd (II) complex was 1 μg/mL BV/0.85 μM Pd (II) complex after 24 hours of incubation. The optimal dose and treatment time of BV alone and in combination with Pd (II) complex to be used in subsequent experiments were set according to Cc_50_ values of these components at 24 hours.

### Cellular morphological changes with BV and BV/Pd (II) complex

**Figure 2:**
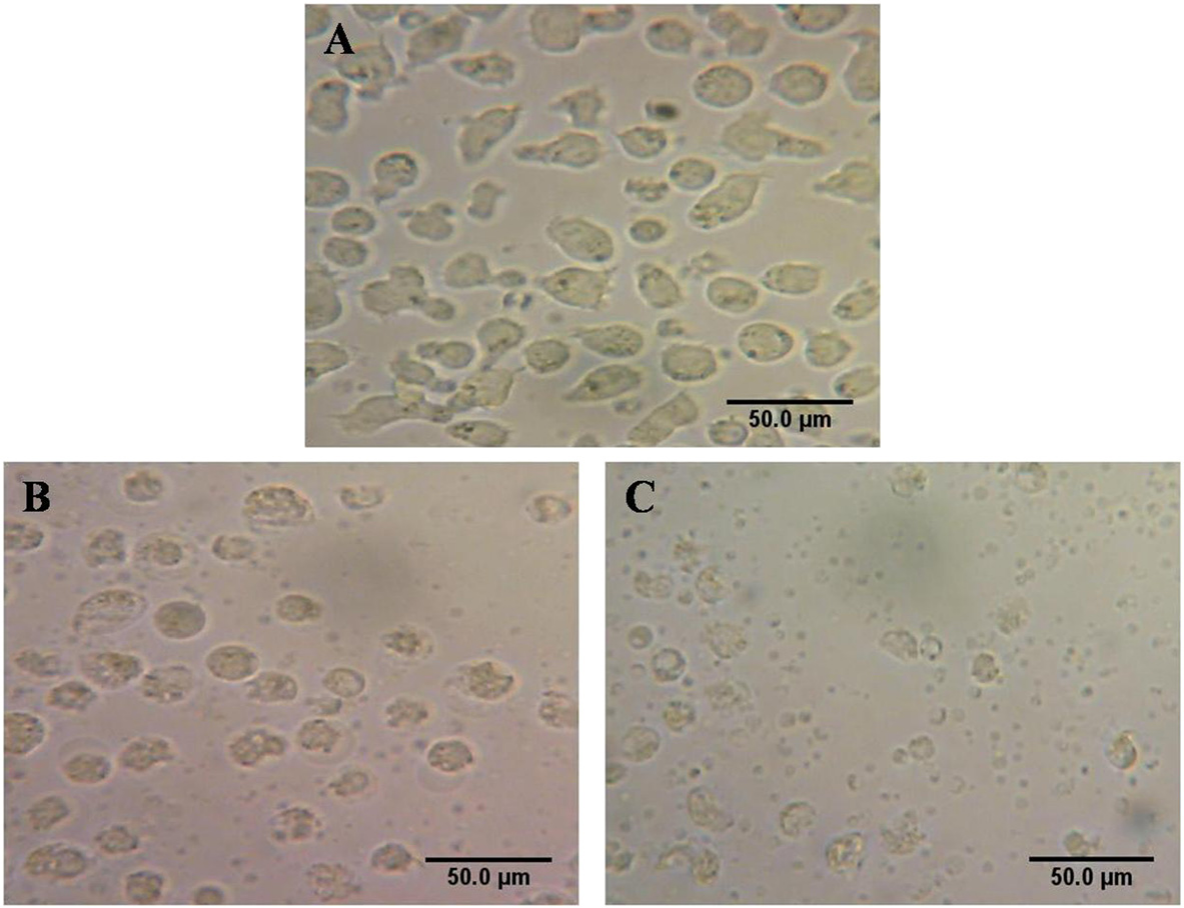
**Effect of BV and BV/Pd (II) complex on the morphology of MOLT-4 cells.** Photomicrographs from inverted microscope. Condensed nuclei obviously indicate apoptosis. **(A)** Controls, **(B)** cell treated with BV, **(C)** cells treated with BV/Pd (II) complex.

To examine the effects of BV and BV/Pd(II) complex on MOLT-4 cell morphology, cells were treated with BV and BV/Pd (II) complex and examined by phase-contrast microscopy. As shown in Figure 2, cells treated with BV (Figure 2B) or with BV/Pd complex (Figure 2C) displayed greater nuclear condensation than the control group (Figure 2A). This morphological characteristic suggests that BV alone or in combination with Pd (II) complex induces apoptotic cell death in MOLT-4 cells.

### Flow cytometry

**Figure 3:**
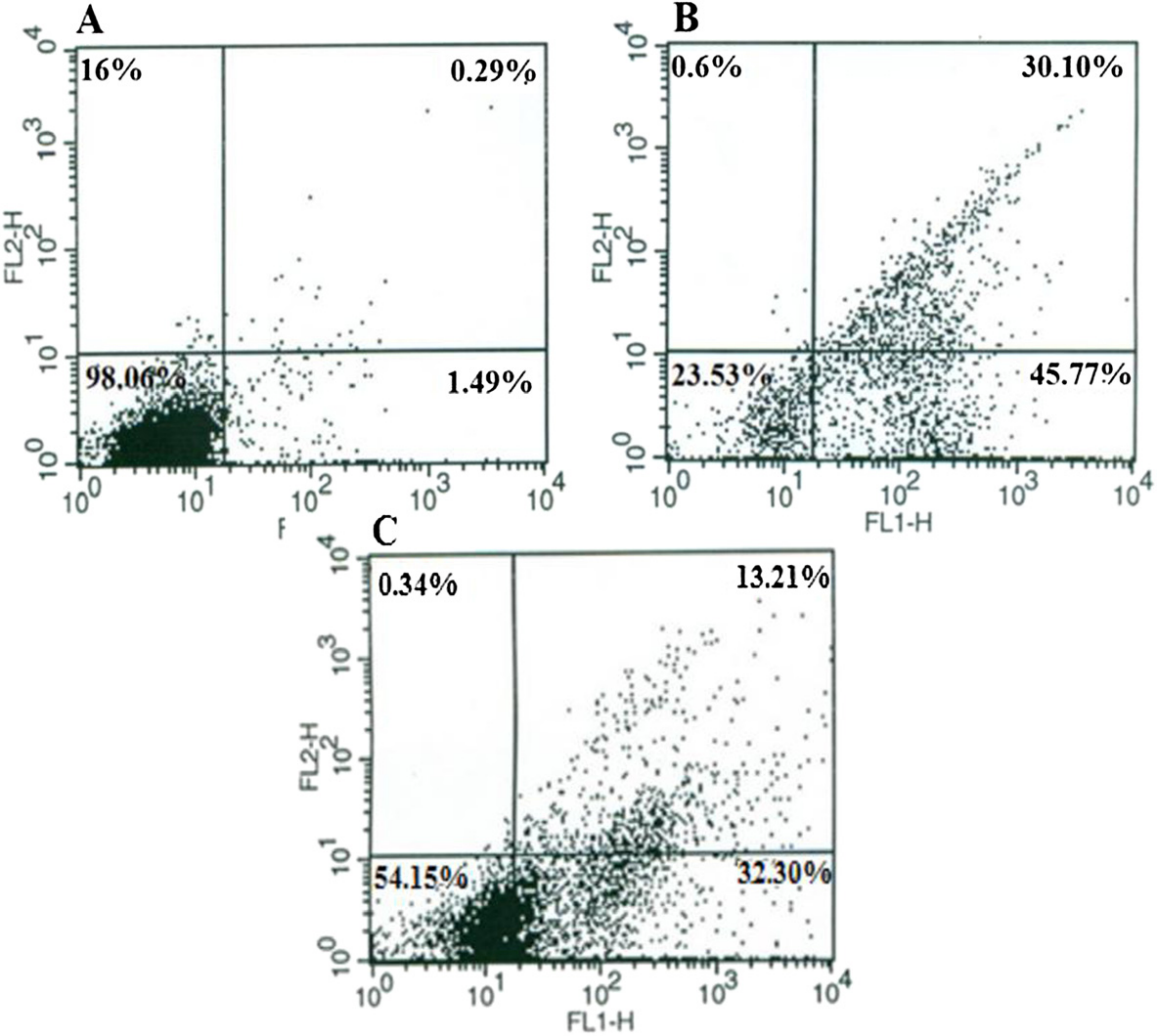
**Characterization of BV and BV/Pd (II) complex-induced apoptosis in MOLT-4 cells by flow cytometry.** Cells were cultured **(A)** without any component (control), **(B)** with BV and **(C)** with BV/Pd (II) complex for 24 hours. Note that 32.30% of the cells exposed to Cc_50_ value of these two components simultaneously [1 μg/mL BV/0.85 μM Pd (II) complex] enter early apoptosis stage.

To prove that BV and BV/Pd(II) complex induce apoptosis in MOLT-4 cells, a flow cytometric analysis with Annexin-V was performed (Figure 3). The results confirmed that the cells exposed to BV alone or in combination with Pd (II) complex for 24 hours enter the early stage of apoptosis. Apoptosis was induced in 32.30% of the cells exposed simultaneously to the Cc_50_ value of these two components.

### Caspase-3 enzyme activity

**Figure 4:**
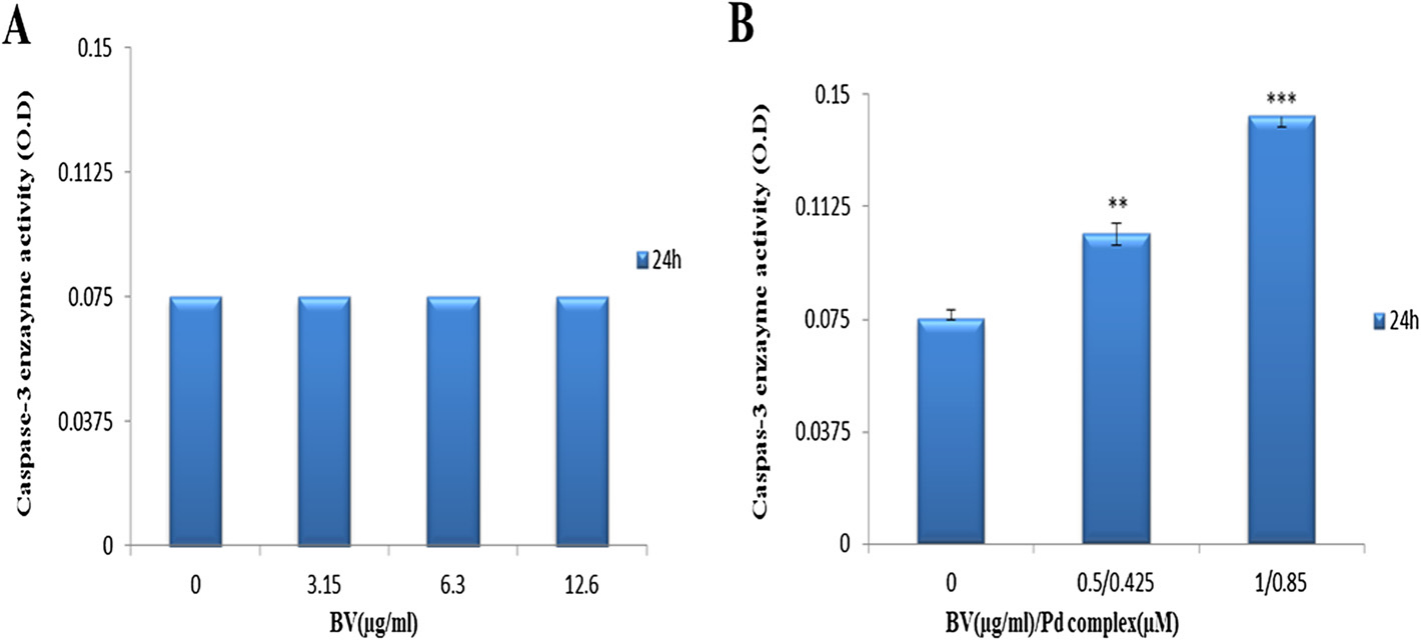
**Results of caspase-3 enzyme activity assay.** Cells treated **(A)** with BV and **(B)** with BV/Pd (II) complex. The optical density was measured at 405 nm. The OD values were not altered by increasing BV, while the OD values rose following a dose increase from 1/2Cc_50_ BV/Pd (II) complex [0.5 μg/mL BV/0.425 μM Pd (II) complex] to Cc_50_ BV/Pd (II) complex [1 μg/mL BV/0.85 μM Pd (II) complex] compared to the control. Data represent mean ± SEM of three different experiments. n = 3; *** p > 0.001 significantly different from the control.

Caspase-3 enzyme activity was measured by a colorimetric assay. The enzyme activity assay revealed that caspase 3 was not affected by BV. The optical density of the samples exposed simultaneously to BV and Pd (II) complex increased from 0.075 to 0.1033 and from 0.075 to 0.14266 at 1/2 Cc_50_ [0.5 μg/mL BV/0.425 μM Pd (II) complex] and Cc_50_ [1 μg/mL BV/0.85 μM Pd(II) complex], respectively (Figure 4).

## Discussion and conclusions

Although it has been previously reported that bee venom can inhibit human cancer cell growth through induction of apoptosis in many cancer cell lines such as prostate cancer, breast cancer and melanoma, there is no finding of the induction of apoptosis in human T cell acute lymphoblastic leukemia cells by BV [6-8]. Based on our knowledge, the present study is the first report about examination of the synergistic effect of BV with a palladium metal-based component.

Analysis of cytotoxicity by MTT assay proved that BV is both time- and dose-dependent in its cytotoxic effects, given that the Cc_50_ values of this component were 6.3 and 0.6 μg/mL after 24 and 48 hours, respectively. Due to the inconsistency in the MTT assay data – as a result of precipitation of the BV at high concentrations after 24 and 48 hours, and at low concentrations after 48 hours – only the concentrations less than 10 μg/mL were applied in these experiments. At these concentrations the findings were acceptable, except at 8 μg/mL after 48 hours, which again resulted in precipitation of the BV.

The lethal dosage of BV in MOLT-4 cells is about 6.3 μg/mL, which is lower than that for lung cancer cells reported by Jang et al. [9] (10 μg/mL), but exceeds that of leukemia U937 cells (about 2 μg/mL after 48 hours), as well as human melanoma A2058 cells (2 μg/mL after one hour) [8,10]. However, BV required different durations to induce cell death in these distinct types of cancerous cells. Such differences may be due to the biological and genetic variations between the investigated cell types. Morphological analysis and the results of flow cytometry indicated that the type of cell death induced by BV is apoptosis.

The present data have also revealed that expression of caspase-3 protein in MOLT-4 cells exposed to BV is down-regulated. Ip et al. [11], when examining the effect of honey bee venom on human cervical epidermoid carcinoma Ca Ski cells, observed that bee venom induced cell cycle arrest and apoptosis in these cells in caspase-dependent and caspase-independent pathways [11]. Tu et al. [8] also indicated that bee venom induces calcium-dependent but caspase-independent apoptotic cell death in human melanoma A2058 cells.

On the contrary, BV induced apoptosis in human leukemia U937 cells through down-regulation of the ERK and Akt signaling pathway, with Bcl-2 and caspase-3 as the key regulators [10]. A large amount of evidence indicates that apoptosis-induced factors (AIF) and endonuclease G (EndoG) act as major apoptosis agents in the caspase-independent cell death pathway [25-28].

In addition to the abovementioned effects, we proved that lethal effects of Pd complex were potentiated by adding a non-lethal dose of the bee venom. On the other hand, BV exerts a strong synergistic effect on the Pd (II) complex. Our preliminary data, which were presented at the FAOBMB Conference, indicated that 1.7 μM [Pd(bpy)(Pi-Pydtc)]NO_3_ produces a cytotoxic effect on the MOLT-4 cells. It was also demonstrated that the lethal dose of this newly synthesized palladium complex can induce apoptosis in these cells [22].

In the present study, we demonstrated that when BV and palladium (II) complex were consumed simultaneously, the combination of 1 μg/mL BV with 0.85 μM Pd (II) complex induces MOLT-4-cell apoptosis in a caspase-3-dependent manner. Orsolic [29], while investigating cytotoxic effects of bee venom applied alone or in combination with the DNA-damaging drug bleomycin on HeLa and V79 cells, found that bleomycin caused a dose-dependent decrease in cell survival. When used with a non-lethal dose of the BV, its lethal effect was potentiated. The author inferred that BV, by preventing repair of damaged DNA, increases bleomycin lethality and inhibited recovery from bleomycin-induced damage [29].

Because DNA is the main target of palladium metal-based complexes, we may conclude that BV is able to potentiate the lethality effect of [Pd (bpy)(Pi-Pydtc)]NO_3_ in this manner. In summary, the results of the present study suggest that the BV induces apoptosis in human lymphoblastic leukemia cells and, if further studies on animal models confirm these results, that bee venom may be used with customary chemotherapy agents to improve their cytotoxic effects.

### Ethics committee approval

The present study was approved by the Ethics Committee of the Faculty of Biological Sciences at Kharazmi University.

## Competing interests

The authors declare that there are no competing interests.

## Authors’ contributions

All authors contributed equally to this work. All authors read and approved the final manuscript.

## References

[B1] Dotimas EM Hider RC Honeybee venom Bee World 1987 19 2 51 70

[B2] Son DJ Lee JW Lee YH Song HS Lee CK Hong JT Therapeutic application of anti-arthritis, pain-releasing, and anti-cancer effects of bee venom and its constituent compounds Pharmacol Ther 2007 19 2 246 270 10.1016/j.pharmthera.2007.04.004 17555825

[B3] Vento R D’Alessandro N Giuliano M Lauricella M Carabillò M Tesoriere G Induction of apoptosis by arachidonic acid in human retinoblastoma Y79 cells: involvement of oxidative stress Exp Eye Res 2000 19 4 503 517 10.1006/exer.1998.0810 10865999

[B4] Winder D Gunzburg WH Erfle V Salmons B Expression of antimicrobial peptides has an antitumour effect in human cells Biochem Biophys Res Commun 1998 19 3 608 612 10.1006/bbrc.1997.8014 9464264

[B5] Kaiser T Brennecke SP Moses EK Methylenetetrahydrofolate reductase polymorphisms are not a risk factor for preeclampsia/eclampsia in Australian women Gynecol Obstet Invest 2000 19 2 100 102 10.1159/000010291 10965192

[B6] Park MH Choi MS Kwak DH Oh KW Yoon do Y Han SB Song HS Song MJ Hong JT Anti-cancer effect of bee venom in prostate cancer cells through activation of caspase pathway via inactivation of NF-kB Prostate 2011 19 8 801 812 10.1002/pros.21296 21456063

[B7] Ip SW Liao SS Lin SY Lin JP Yang JS Lin ML Chen GW Lu HF Lin MW Han SM Chung JG The role of mitochondria in bee venom-induced apoptosis in human breast cancer MCF7 cells In Vivo 2008 19 2 237 245 18468409

[B8] Tu WC Wu CC Hsieh HL Chen CY Hsu SL Honeybee venom induces calcium-dependent but caspase-independent apoptotic cell death in human melanoma A2058 cells Toxicon 2008 19 2 318 329 10.1016/j.toxicon.2008.06.007 18602939

[B9] Jang MH Shin MC Lim S Han SM Park HJ Shin I Lee JS Kim KA Kim EH Kim CJ Bee venom induces apoptosis and inhibits expression of cyclooxygenase-2 mRNA in human lung cancer cell line NCI-H1299 J Pharmacol Sci 2003 19 2 95 104 10.1254/jphs.91.95 12686753

[B10] Moon DO Park SY Heo MS Kim KC Park C Ko WS Choi YH Kim GY Key regulators in bee venom-induced apoptosis are Bcl-2 and caspase-3 in human leukemic U937 cells through downregulation of ERK and Akt Int Immunopharmacol 2006 19 12 1796 1807 10.1016/j.intimp.2006.07.027 17052670

[B11] Ip SW Wei HC Lin JP Kuo HM Liu KC Hsu SC Yang JS Chiu TH Han SM Chung JC Mei-Dueyang Bee venom induced cell cycle arrest and apoptosis in human cervical epidermoid carcinoma Ca Ski cells Anticancer Res 2008 19 2A 833 842 18507026

[B12] Orsolic N Bee venom in cancer therapy Cancer Metastasis Rev 2012 19 1–2 173 194 22109081 10.1007/s10555-011-9339-3

[B13] Kaufmann SH Earnshaw WC Induction of apoptosis by cancer chemotherapy Exp Cell Res 2000 19 1 42 49 10.1006/excr.2000.4838 10739650

[B14] Hengartner MO The biochemistry of apoptosis Nature 2000 19 6805 770 776 10.1038/35037710 11048727

[B15] Cohen GM Caspases: the executioners of apoptosis Biochem J 1997 19 Pt 1 1 16 9337844 10.1042/bj3260001 PMC1218630

[B16] Sun RW Ma DL Wong EL Che CM Some uses of transition metal complexes as anti-cancer and anti-HIV agents Dalton Trans 2007 19 43 4884 4892 17992273 10.1039/b705079h

[B17] Cooley ME Davis LE DeStefano M Abrahm J Cisplatin: a clinical review. Part I - Current uses of cisplatin and administration guidelines Cancer Nurs 1994 19 3 173 184 8055487

[B18] Martínez A Lorenzo J Prieto MJ Font-Bardia M Solans X Avilés FX Moreno V Influence of the position of substituents in the cytotoxic activity of trans platinum complexes with hydroxymethylpyridines Bioorg Med Chem 2007 19 2 969 979 10.1016/j.bmc.2006.10.031 17088064

[B19] Ronconi L Giovagnini L Marzano C Bettìo F Graziani R Pilloni G Fregona D Gold dithiocarbamate derivatives as potential antineoplastic agents: design, spectroscopic properties, and *in vitro* antitumor activity Inorg Chem 2005 19 6 1867 1881 10.1021/ic048260v 15762713

[B20] Akdi K Vilaplana RA Kamah S Navarro JA Salas JM González-Vílchez F Study of the biological effects and DNA damage exerted by a new dipalladium-Hmtpo complex on human cancer cells J Inorg Biochem 2002 19 1–2 51 60 12009255 10.1016/s0162-0134(02)00370-7

[B21] Mansoori-Torshizi H Islami-Moghaddam M Saboury AA A microcalorimetry and spectroscopy study on the interaction of BSA with 2, 2’-bipyridine octylglycinato Palladium (II) Nitrate Acta Biochim Biophys Sin (Shangai) 2003 19 10 886 890 14515204

[B22] Nabiuni M Divsalar A Mansouri-Torshizi H Safayinejad Z Cytotoxic effect of honey bee venom and novel palladium complex against human T- cell acute lymphoblastic cell line (MOLT4) [abstract] Proceedings of FAOBMB Conference 2011 Singapore

[B23] Lariviere WR Melzack R The bee venom test: a new tonic-pain test Pain 1996 19 2–3 271 277 8880850 10.1016/0304-3959(96)03075-8

[B24] Mansouri-Torshizi H Eslami-Moghadam M Divsalar A Saboury AA DNA-binding studies of some potential antitumor 2, 2’–bipyridine Pt (II)/Pd (II) complexes of piperidinedithiocarbamate. Their synthesis, spectroscopy and cytotoxicity Acta Chim Slov 2011 19 811 822 24061133

[B25] Joza N Susin SA Daugas E Stanford WL Cho SK Li CY Sasaki T Elia AJ Cheng HY Ravagnan L Ferri KF Zamzami N Wakeham A Hakem R Yoshida H Kong YY Mak TW Zuniga-Pflucker JC Kroemer G Penninger JM Essential role of the mitochondrial apoptosis-inducing factor in programmed cell death Nature 2001 19 6828 549 554 10.1038/35069004 11279485

[B26] Li LY Luo X Wang X Endonuclease G is an apoptotic DNase when released from mitochondria Nature 2001 19 6842 95 99 10.1038/35083620 11452314

[B27] Miramar MD Costantini P Ravagnan L Saraiva LM Haouzi D Brothers G Penninger JM Peleato ML Kroemer G Susin SA NADH oxidase activity of mitochondrial apoptosis-inducing factor J Biol Chem 2001 19 19 16391 16398 10.1074/jbc.M010498200 11278689

[B28] Kroemer G Martin SJ Caspase-independent cell death Nat Med 2005 19 7 725 730 10.1038/nm1263 16015365

[B29] Orsolic N Potentiation of Bleomycin lethality in HeLa and V79 cells by bee venom Arh Hig Rada Toksikol 2009 19 3 317 326 19789161 10.2478/10004-1254-60-2009-1936

